# In Reversion

**Published:** 1892-04-30

**Authors:** 


### Passing Topics.

### IN REVERSION.

It is said that the element of uncertainty adds the chief zest to the pleasure of hope, and without doubt we are prone to set a high value upon such things as we are not altogether certain of obtaining. But we must not push the paradox. Most people, for instance, would admit a preference for the bequest absolute over that which is reversionary, and are apt to regard the latter as an incomplete and rather unsatisfactory evidence of the testator's interest and affection. Annuitants are proverbially long-lived, and the actual beneficiary occasionally persists in surviving the one who is only expectant, while even if this climax be not reached, there are long years of waiting with feelings so mixed as to afford a perpetual and irritating problem to their victim, who seldom has the candour to admit, even to himself, that

"I've dearly loved my uncle John,
From childhood to the present hour,
And yet he will go living on,
I would he were a tree or flower."

It is reassuring to reflect that although individuals may suffer, institutions, however philanthropic in character, are yet insentient organisations, else what would be the sensations of the Brompton Hospital for Consumption upon receiving notice of a legacy of £200 Consols left in trust for its ultimate benefit, but subject to the use of the income for keeping in order the testator's family grave in Brompton Cemetery "for twenty years and six months after the death of the last survivor of the descendants (living at the time of the testator's decease) of Queen Victoria"? What a magnificent combination, first of the precise with the indefinite—twenty years and six months after unreckoned ages—and then of the regal with the mortal—Princes, Palaces, and a grave in a London cemetery.

Mark how robust is the faith of the testator in the imperishability of the Brompton Hospital, consumption and consols, as compared with the weaker belief in the permanence of the cemetery. To some less conscious of the immutability of first principles, the thought might have occurred that if it were desired to benefit the hospital the gentleman in question clearly was no believer in the but better to assist while the necessities were apparent, proverb, *bis dat qui cito dat*. On the contrary, he must have thought that generosity improves by keeping, and it will be an interesting calculation, upon which the Secretary may profitably employ his leisure moments, to determine when the legacy is likely to become receivable and what will then be its net value?

We may be permitted to hope that this testator's eccentricity will not prove attractive to others. If it becomes customary to load bequests to hospitals in the particular way chosen in the instance under notice, or indeed, in any similar fashion, hospitals will acquire a real grievance. It was once put forward as recommendatory of a suggested site for a sick asylum that it was conveniently placed for the cemetery, and from the fact that the building was ultimately erected there, colour was given to the assertion that the suggestion had not been without its effect. The Brompton Cemetery is a famous institution as we all know, but we think it would have been kinder to have spared the hospital the reflection that it held anything in common with its neighbour. Upon the whole it is better to encourage the old-fashioned idea that hospitals are aids to health rather than to dissolution. Agricola.

